# Inspection of Defective Glass Bottle Mouths Using Machine Learning

**DOI:** 10.3390/jimaging11040105

**Published:** 2025-03-29

**Authors:** Daiki Tomita, Yue Bao

**Affiliations:** Setagaya Campas, Tokyo City University, Tokyo 158-8557, Japan; bao@tcu.ac.jp

**Keywords:** image processing, machine learning, chip inspection, glass bottles

## Abstract

In this study, we proposed a method for detecting chips in the mouth of glass bottles using machine learning. In recent years, Japanese cosmetic glass bottles have gained attention for their advancements in manufacturing technology and eco-friendliness through the use of recycled glass, leading to an increase in the volume of glass bottle exports overseas. Although cosmetic bottles are subject to strict quality inspections from the standpoint of safety, the complicated shape of the glass bottle mouths makes automated inspections difficult, and visual inspections have been the norm. Visual inspections conducted by workers have become problematic because it has become clear that the standard of judgment differs from worker to worker and that inspection accuracy deteriorates after long hours of work. To address these issues, the development of inspection systems for glass bottles using image processing and machine learning has been actively pursued. While conventional image processing methods can detect chips in glass bottles, the target glass bottles are those without screw threads, and the light from the light source is diffusely reflected by the screw threads in the glass bottles in this study, resulting in a loss of accuracy. Additionally, machine learning-based inspection methods are generally limited to the body and bottom of the bottle, excluding the mouth from analysis. To overcome these challenges, this study proposed a method to extract only the screw thread regions from the bottle image, using a dedicated machine learning model, and perform defect detection. To evaluate the effectiveness of the proposed approach, accuracy was assessed by training models using images of both the entire mouth and just the screw threads. Experimental results showed that the accuracy of the model trained using the image of the entire mouth was 98.0%, while the accuracy of the model trained using the image of the screw threads was 99.7%, indicating that the proposed method improves the accuracy by 1.7%. In a demonstration experiment using data obtained at a factory, the accuracy of the model trained using images of the entire mouth was 99.7%, whereas the accuracy of the model trained using images of screw threads was 100%, indicating that the proposed system can be used to detect chips in factories.

## 1. Introduction

In recent years, Japanese cosmetic glass bottles have garnered significant attention due to advancements in manufacturing technology and their eco-friendly attributes, particularly through the use of recycled glass [1]. Consequently, the export volume of these glass bottles has been increasing [2].

**Figure 1:**
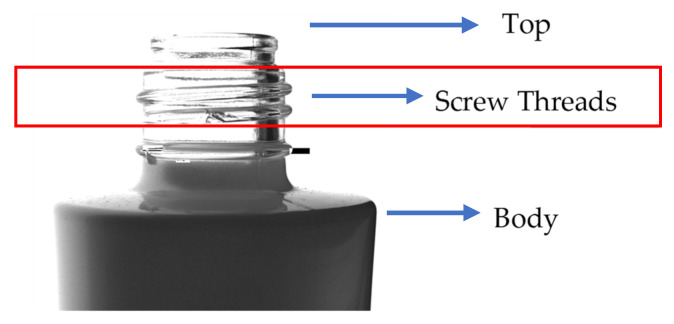
Parts of a glass bottle and an example of the chipping focused on in this study.

Cosmetic glass bottles are considered high-end products and are therefore subjected to stringent quality inspections. Notably, their necks are often designed with screw threads for capping, and the complexity of these structures frequently results in manufacturing defects. Moreover, previous studies have actively focused on detecting chipping at the top and body of glass bottles; however, chipping in the threaded section has not been extensively investigated [3,4]. Figure 1 illustrates the nomenclature of each part of the glass bottle, their respective positions, and an image depicting chipping that occurs in the threaded region.

In visual inspections, where defective products are assessed by human operators, judgment criteria may vary among individuals. Furthermore, a study by Hida et al. demonstrated that sustained attention declines during prolonged inspection tasks, leading to a 5–10% reduction in inspection accuracy after just five consecutive trials [5]. This calls for the development of inspection equipment that uses image processing to improve productivity. This paper proposes a method for acquiring screw threads at the mouth of glass bottles and a machine learning-based approach for detecting chips using these screw threads. The proposed method is expected to enhance productivity in manufacturing environments.

## 2. Related Work

In glass bottle manufacturing plants that operate on a mass-production scale, there is a critical need for inspection equipment capable of conducting highly accurate, non-destructive, and non-contact assessments throughout the production process. Typically, camera-based inspection systems integrated along the conveyor line are the standard approach in such facilities. The following is a list of related studies.

### 2.1. General Image Processing Methods [3,6,7]

In a study by Huimin Ma et al., a 360-degree inspection of a bottle’s sides and an examination of its top surface were conducted using six cameras for the sides and two cameras for the top surface [3]. For chip detection, the captured image is first converted to grayscale, followed by dynamic correction of luminance values. This correction is performed by comparing the average luminance value of the pixel of interest with that of neighboring pixels. If the pixel of interest has a higher luminance value than the average, it is replaced with the smallest luminance value in the neighborhood, as it may indicate a missing region. Subsequently, luminance values are adjusted by computing the difference between the corrected pixel and its original value, extracting only areas with a high likelihood of missing pixels. The extracted image is then binarized to detect chips. However, since this method was designed for beverage bottles without screw threads, it cannot be applied to glass bottles with screw threads due to variations in light reflection properties. Consequently, this approach is unsuitable for the glass bottles examined in this study.

A patented technique for glass bottle inspection process is the use of a ring-shaped inspection gate [6]. In this method, a camera and a dome-shaped light source are installed on top of the glass bottle to take pictures. By shooting from directly above the glass bottle, the top surface of the bottle and the upper part of the screw threads appear as a ring in the shot image. The photographed image is then searched for areas that exceed an arbitrary shade difference, and these areas are detected as chips or scratches. With this method, the top surface and the upper part of the screw thread can be inspected normally because the image is taken from the top. However, if chips exist in the lower part of the screw thread or in the second round of the screw thread, the chips cannot be detected.

The authors proposed a method for detecting chipping on a 180-degree thread on the half face of a bottle using an infrared surface light source and three infrared cameras [7]. This method was shown to be able to inspect a screw thread area of 180 degrees on the half face of the bottle with an accuracy of approximately 99%. However, there are problems with this method: the chipping threshold is determined based on known chipping, so it cannot handle unknown chipping, and the parameters for determining the inspection area are set in detail, so it is difficult to apply the method to other types of bottles.

### 2.2. Classification Tasks Using Machine Learning [4,8,9]

Over recent years, significant research has been conducted on inspection systems utilizing machine learning, driven by advancements in GPU precision.

Image classification tasks using machine learning include sorting fruit types [8] and detecting defects in cast parts [9].

**Figure 2:**
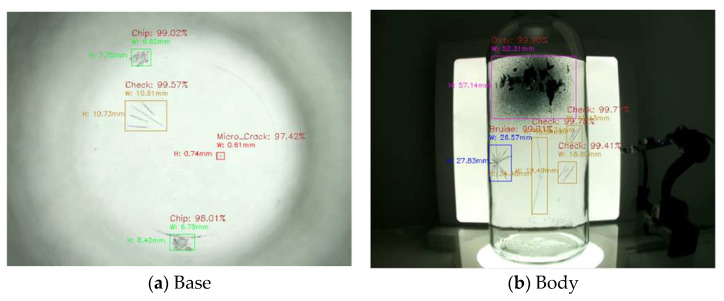
Scope of inspection in conventional research (inspection of bottle bottom (**a**) and bottle body (**b**)).

In a study related to glass bottles inspection, M.A.E. Latina et al. have conducted a study on defect detection in empty glass bottles based on deep learning with CNN using the MobileNetV2 model [4]. This method leverages machine learning to detect various defects, ranging from large scratches to small chips and stains, on the sides and bottoms of empty bottles. As a result, defective bottles can be identified with a high accuracy of 98%. In addition, this approach utilizes two Raspberry Pi devices for integration into an industrial application and employs the lightweight MobileNetV2 model. However, the inspection is limited to the bottle’s body and bottom, as shown in Figure 2, and is not applicable to the mouth, which features a more complex geometry.

## 3. Proposed Method

**Figure 3:**
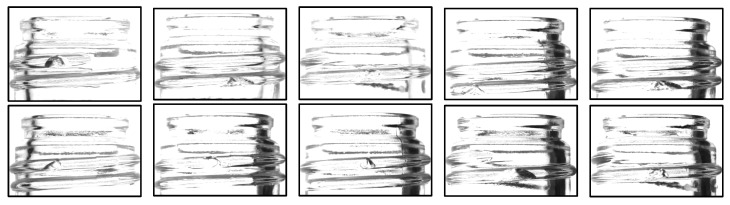
Examples of deficiencies covered in this study (Chips are present on the threads of all bottles).

This study proposes an inspection method capable of detecting chips in the mouths of glass bottles with complex shapes. An infrared camera and an infrared backlight are utilized for image acquisition. The captured images are then processed and used as a dataset for a machine learning model, which classifies them into two categories: “chipped” and “not chipped.” The main objective of this study is to detect cracks and chips of about 5 mm that occur on threads as “chips”. An image showing examples of chips that occur is shown in Figure 3.

### 3.1. Capturing Bottle Images

**Figure 4:**
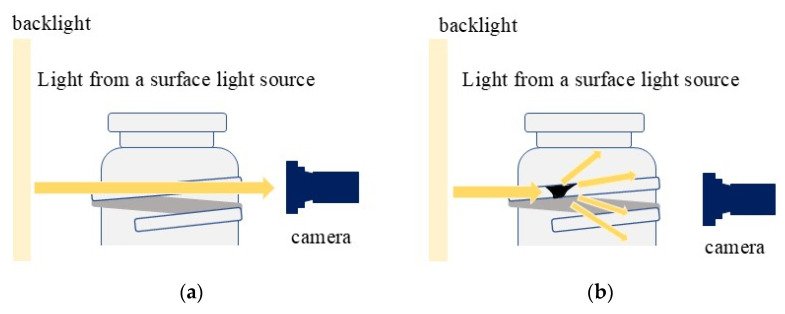
Overview of glass bottle photography. (If there are no chips, light passes through intact (**a**). When there are chips, light reflects diffusely through the chips, resulting in a dark reflection (**b**)) [7].

The method previously used by the authors is used for filming [7]. The glass bottles used in this study are transparent, so when the glass bottles are photographed through an infrared surface light source, light penetrates the smooth surface of the glass bottles without chips as shown in Figure 4 and the image appears white. However, if there are chips or scratches on the glass surface, the light is diffusely reflected on the surface, making the area appear darker. This property is used to detect chips and scratches. In addition, a cut filter is used to shield the light from ambient light. By shading ambient light, inspections can be performed without being affected by lighting in the factory or inspection area.

### 3.2. Configuration of the Proposed System

**Figure 5:**
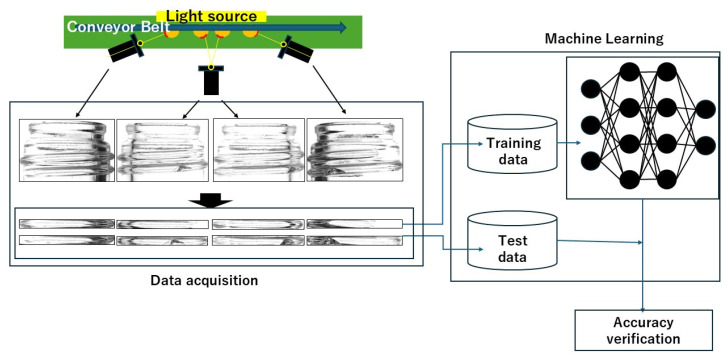
Configuration of the proposed system.

Figure 5 illustrates the proposed system for this study. First, an infrared camera captures images of glass bottles as they move along a conveyor. The screw thread regions are extracted from the captured images and used to train the model, with separate datasets for training and testing. The accuracy of the model is then evaluated based on the test data.

### 3.3. Image Preprocessing

**Figure 6:**
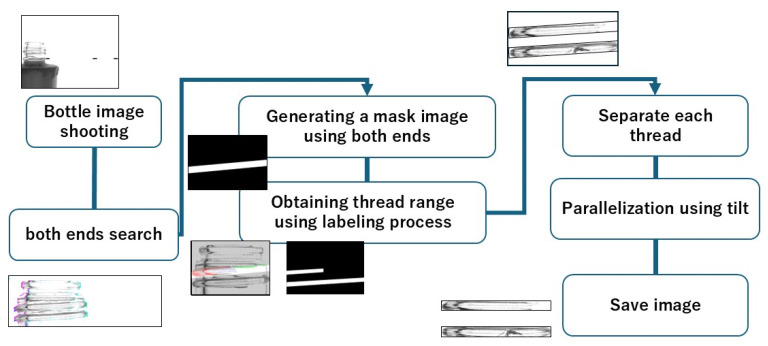
Pre-processing flowchart.

As a pre-processing step, an image of only the screw threads is acquired from the captured image. Figure 6 shows the acquisition flow. Image preprocessing was conducted to obtain parallel screw thread images. This process included acquiring thread coordinates, generating mask images, and performing labeling, utilizing methods previously developed by the authors [7].

#### 3.3.1. Detection of the Screw Thread Endpoints [7]

**Figure 7:**
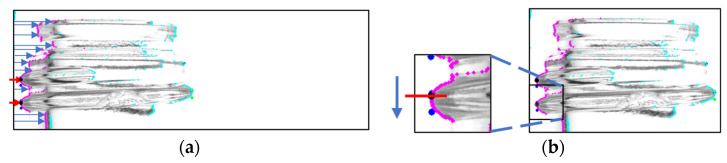
Process to search both ends of the screw threads. (A process that searches both ends (**a**) and a process that further searches the screw thread region (**b**)) [7].

To extract the screw threads, the endpoints of the glass bottle were identified. Figure 7a illustrates the search process from the left side. The pixel with the lowest luminance value corresponding to the bottle’s contour was retained, and based on its coordinate information, the leftmost point and the second leftmost point were acquired as the endpoints of the screw threads. The top and bottom boundaries of the screw threads were determined by scanning a few additional pixels above and below these retained points, as shown in Figure 7b.

In the figure, the magenta and cyan dots represent the outline coordinates of the scanned glass bottle, the black dots indicate the retained coordinates of the screw thread endpoints, and the dark blue and green dots denote the top and bottom boundaries of the screw threads identified through this process. The blue arrows in A in the figure show the scanning and the arrows in B show the search area.

#### 3.3.2. Mask Generation and Screw Thread Range Detection [7]

**Figure 8:**
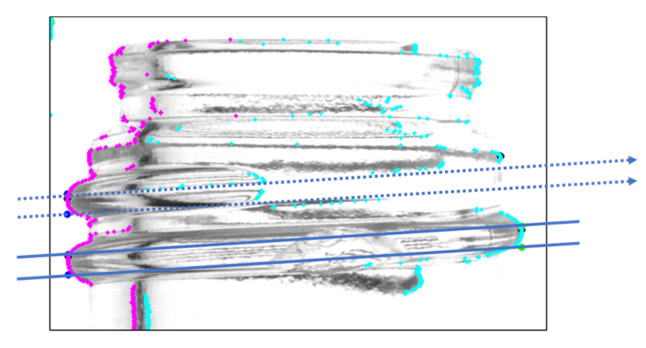
Screw thread range depicted when both endpoints are successfully identified (solid line) [7].

If there are threads at both ends based on the obtained contour information, the thread range can be obtained, as shown by the solid line. However, if the screw threads are lost in the middle (upper example in Figure 8), the coordinate information on one side is available but not the other side, so the range of the screw threads cannot be accurately obtained as shown by the dotted line.

**Figure 9:**
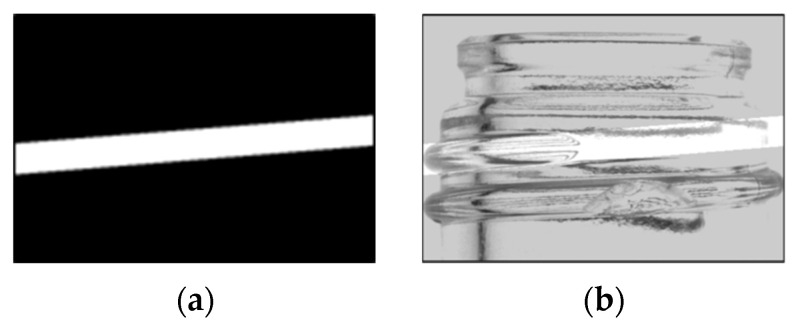
Generated mask image (**a**) and the resulting composite image (**b**) [7].

Therefore, a mask image, as shown in Figure 9, was generated to accurately define the extent of the screw threads. The mask image was designed with sufficient thickness to fully cover the top and bottom of the threads, and the thread region was identified by detecting continuous shadow areas present above and below the screw threads. The slope of the mask image was determined based on the inclination of the threads.

#### 3.3.3. Screw Thread Range Detection Using Labeling [7]

**Figure 10:**
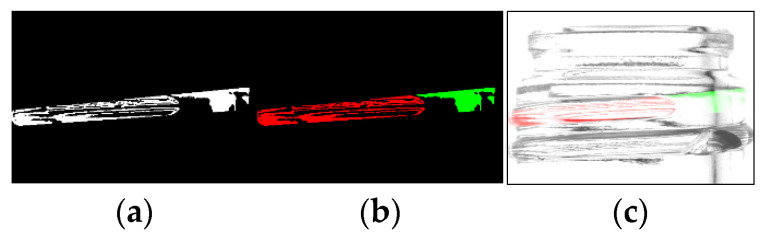
Labeling process using Connected Components with Stats (binarized image (**a**), labeled image (**b**), composite image (**c**)) [7].

The OpenCV “Connected Components With Stats” function [10,11] is used to obtain the extent of missing threads by detecting continuous regions of shadow. The process is shown in Figure 10. The area shown in red in the figure indicates the contiguous area from the left, and the area shown in green indicates the contiguous area from the right.

**Figure 11:**
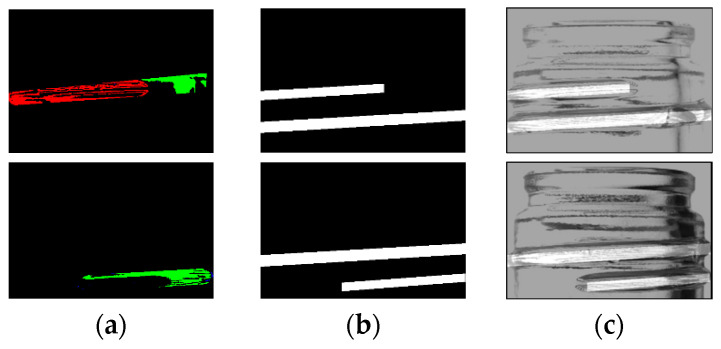
Generated mask and synthesized images. Label image (**a**), mask image (**b**), composite image (**c**) [7].

The label information obtained was then used to generate a mask image that matched the extent of the threads. Figure 11 shows the generated mask image and the composite image.

#### 3.3.4. Rotation Correction

**Figure 12:**
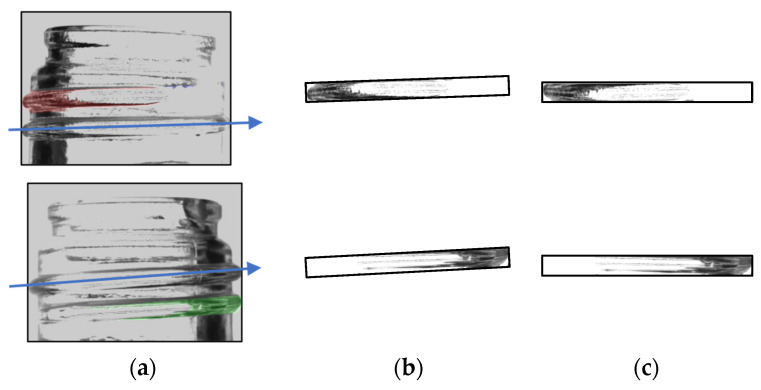
Rotation correction. Composite image of original image and label image (**a**), cropped image (**b**), image with rotation correction (**c**).

Based on the coordinate values obtained by the labeling process, trimming is performed to create a screw thread image. As shown in Figure 12, a parallel thread image is obtained by performing rotation correction based on the tilt of the image created.

### 3.4. Model Selection

In the field of image processing, convolutional neural networks (CNNs) have garnered significant attention due to their high accuracy and economic potential. Common CNN architectures used in image processing and classification include AlexNet [12], VGG16 [13], InceptionV3 [14], ResNet [15], and MobileNetV2 [16].

Convolutional computation plays a crucial role in computer vision tasks. However, due to the deep and complex structure of most networks, processing time and computational costs tend to increase. MobileNetV2, on the other hand, mitigates these challenges by employing an inverse residual structure and a linear bottleneck design, thereby reducing convolutional computation. Additionally, previous studies have demonstrated that MobileNetV2 is memory-efficient and achieves superior accuracy compared to other models [8,9]. Table 1 shows the accuracy of each model presented in previous studies [8].

**Figure 13:**
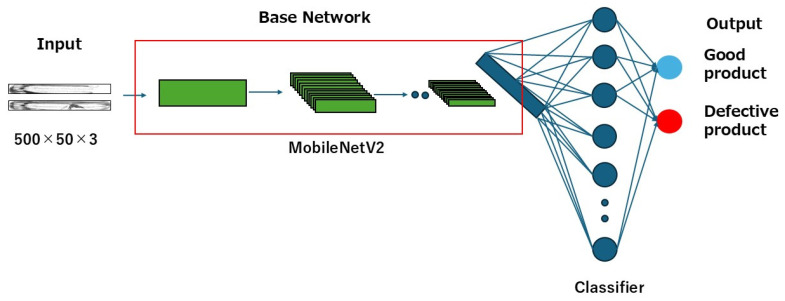
Composition of the learning model.

Based on these advantages, MobileNetV2 architecture was adopted in this study. As hyperparameters, the learning rate was set to 0.00001, the batch size to 16, and the number of epochs to 500; categorical cross-entropy was used as the loss function, and Adam was employed as the optimization method. To avoid over-training the model, an early stopping strategy of 20 epochs was introduced during training. This strategy monitors the performance of the model during training and stops training when signs of overtraining are detected. Figure 13 shows the structure of the training model and Table 2 and Table 3 show the details of the hyperparameters used.

#### Data Argumentation

The number of chipped glass bottles used in this study was small, approximately 40. Since this amount of data may not be sufficient to train a CNN, data augmentation was used to generate a final image with a resolution of 500 × 50 pixels. The data augmentation techniques applied to the dataset are listed in Table 4.

Regarding flipping, this study generates training images for both the entire glass bottle mouth and the screw thread region. Therefore, only left-to-right mirroring was applied to the full mouth image, as vertical inversion could potentially distort the inherent characteristics of the glass bottle. Additionally, even with left-to-right inversion, as shown in Figure 11, a pattern where the screw thread terminates at the midpoint appears at both endpoints. Hence, this transformation is not expected to impact on the learning process.

Regarding rotation, small-angle rotations were applied to preserve the essential characteristics of the glass bottle, particularly when considering the analysis of the entire mouth image.

## 4. Experiments

Two experiments were conducted to demonstrate the effectiveness of this study. In Experiment 1, we confirmed the inspection accuracy of the training model by using the screw thread image dataset, which was extracted from the image of the glass bottle mouth. For comparison, we also confirmed the inspection accuracy of the training model by using the entire mouth image for training. In Experiment 2, we conducted a demonstration experiment to check the inspection accuracy of images taken at an actual factory. The inspection accuracy was evaluated using the indices of fit rate, reproducibility, F value, and correct response rate.

### 4.1. Experimental Environment

**Figure 14:**
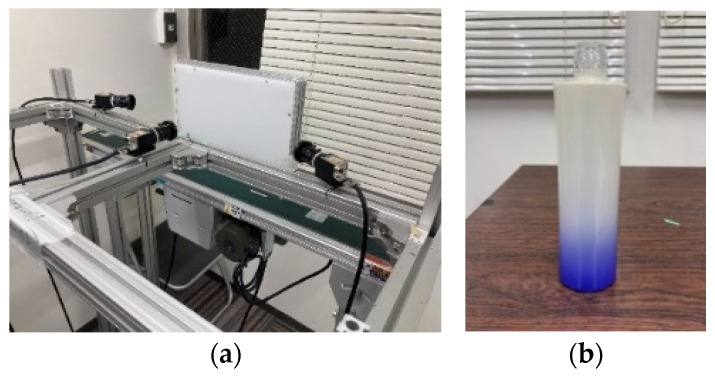
Experimental environment. Experimental setup (**a**) and glass bottle used in the study (**b**).

**Figure 15:**
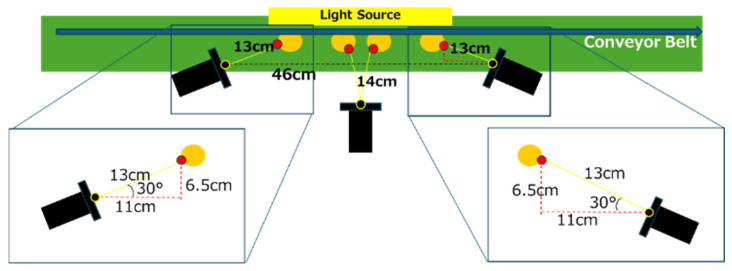
Schematic drawing of the experimental environment [7].

Bottle images were acquired using an experimental environment previously created by the authors [7]. Figure 14 illustrates the experimental environment and the glass bottles used in the study. Table 5 lists the components and their specifications for the experimental setup. In addition, Figure 15 provides a detailed diagram of the camera arrangement. The central camera was positioned 14 cm from the glass bottle, while the left and right cameras were angled at 30° and spaced 13 cm from the glass bottle.

### 4.2. Taking Bottle Images

**Figure 16:**
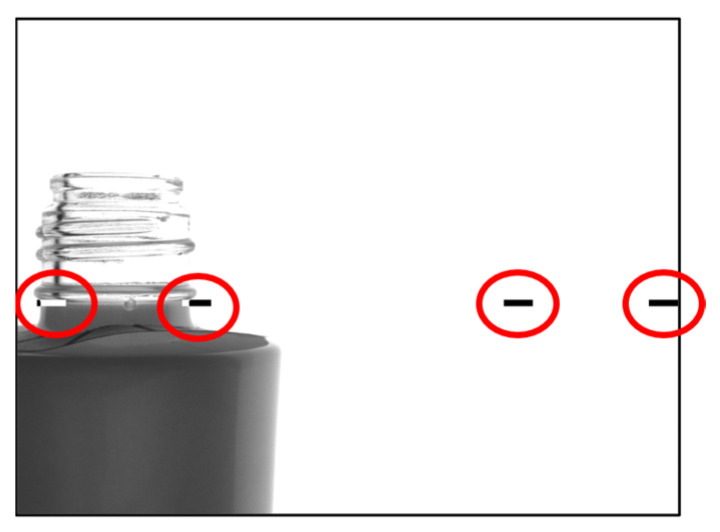
How to take bottle images (taken when a glass bottle is placed in the marker area indicated by the red circle) [7].

In the factory, glass bottles flowing on a conveyor belt are placed by a machine. In the recreated environment, the bottles were placed manually on the conveyor and photographed. The bottles that are swept onto the conveyor look like Figure 16 on the camera. Bottles flowing on the conveyor are photographed with respect to two markers (indicated by red circles) and cut out. In this example, the center camera is shown, which takes two images, one on each side of the figure. We believe that the position of the marker can be changed accordingly when a different type of glass bottle is used, but this study was not conducted with any different types of glass bottles.

### 4.3. Experiment 1

In this experiment, half of the chipped surface was rotated by 180° in 30° increments, and 7 × 4 images were captured and used as training data. A total of 99 bottles (70 defect-free and 29 defective) were prepared for training, while 40 bottles (30 defect-free and 10 defective) were used for validation. The total number of images captured was 938 for defect-free bottles (640 for training and 298 for validation) and 932 for defective bottles (668 for training and 244 for validation). The data were padded as described in Section 3.4 and expanded tenfold for each original image. A training model was developed using both the screw thread and entire mouth images to assess the inspection accuracy. The number of training and test data images is presented for each experiment; however, only the training data images were utilized for model training, with 80% allocated for training and 20% for validation. Thus, no test data were used in the training process.

#### 4.3.1. Screw Thread Image Learning Experiment 1

**Figure 17:**
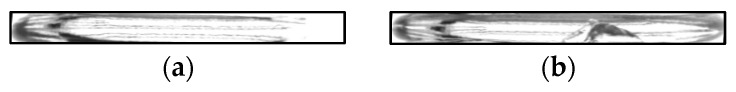
Image used for screw thread learning experiment 1 (good image (**a**), chipped image (**b**)).

Among the 932 defective images, 328 screw threads with chips were identified in the training set and 141 in the validation set. The bottle images used for training is shown in Figure 17, and summarizing the number of images are shown in Table 6.

#### 4.3.2. Experimental Results (Screw Thread)

Accuracy was evaluated using 621 validation images against the model trained on the acquired screw thread images. Table 7 shows the confusion matrix of the detection results.

#### 4.3.3. Mouth Image Learning Experiment 1

**Figure 18:**
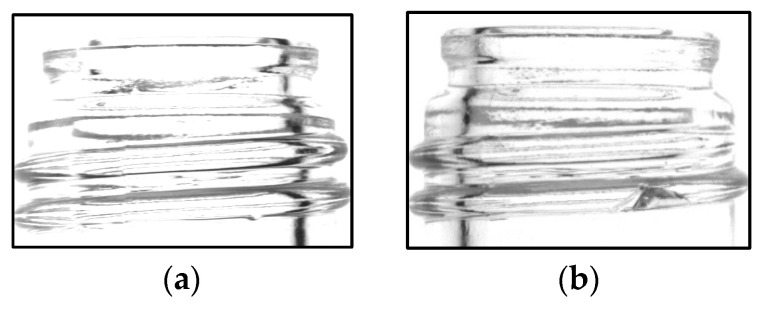
Mouth image used for experiment 1 (good image (**a**), chipped image (**b**)).

For comparison, images of the entire mouth were utilized for training to assess inspection accuracy. The number of defective images obtained was 942. The number of good images was matched to the number of defective images, and the number of images for verification was 298 good and 244 defective images, respectively. The bottle images used for training is shown in Figure 18, and summarizing the number of images are shown in Table 8.

#### 4.3.4. Experimental Results 1 (Mouth of the Bottle Image)

Accuracy was evaluated using 542 validation images against a model trained on images of the entire mouth. Table 9 shows the confusion matrix of the detection results.

#### 4.3.5. Comparison of Experimental Results 1

Table 10 summarizes the inspection accuracy using the entire mouth and the screw threads only.

#### 4.3.6. Discussion of Experimental Results 1

**Figure 19:**
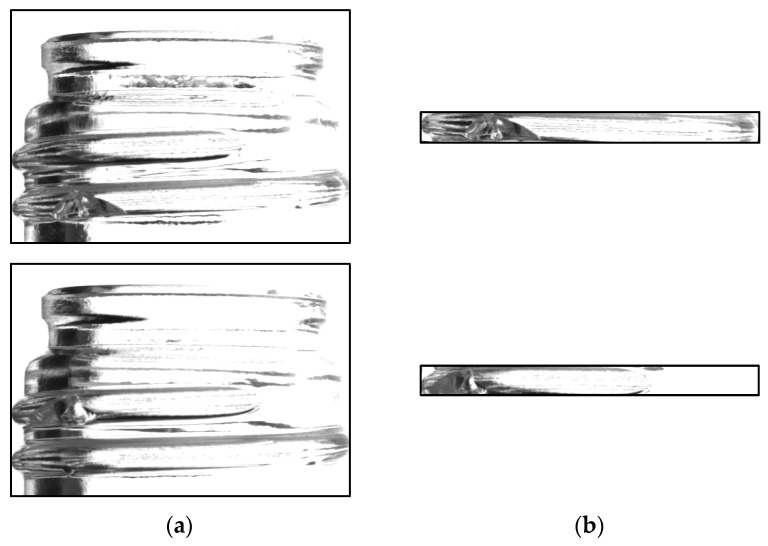
Example of false detection at the entire mouth (**a**) and correct determination at the screw threads (**b**).

Experimental results indicated that the model trained on images of screw threads achieved a detection accuracy of 99.7%, surpassing the 98.0% accuracy of the model trained on images of the entire mouth. In manufacturing, it is crucial to prevent defective products from being misclassified as acceptable. Regarding the recall of defective products, the model trained on thread images achieved a recall of 98.6%, whereas the model trained on entire mouth images had a recall of 95.5%, demonstrating an improvement of approximately 3.1%. Figure 19 illustrates an example of a defective product misclassified as non-defective when evaluated using the entire mouth image yet correctly identified as defective with the screw thread image. This case suggests that false positives are more likely when chips overlap with the glass bottle’s shadow and that limiting the analysis to the screw thread region accentuates the relative area of chipping, thereby improving detection accuracy.

The proposed method was shown to be effective because the model using only screw thread images improved the correctness rate by 1.7% compared to the model in which the entire mouth was trained.

#### 4.3.7. Comparison with Previous Studies

To validate the effectiveness of the proposed method, the results were compared with those of prior studies. The studies referenced for comparison include a glass bottle scratch inspection method using image processing [3,7] and a glass bottle scratch inspection method utilizing machine learning [4]. Table 11 provides a summary of the evaluation of these previous studies and the inspections conducted in this study.

The conventional study utilizing image processing required different threshold values to determine chipping for each bottle. When the threshold was optimized, the inspection accuracy reached 98% in the conventional study [3] (left side of the table) and 99.0% in the conventional study [7] (right side of the table). In comparison, the proposed method eliminates the need to set a threshold for defective bottles, achieving an inspection accuracy of 99.7%, thus demonstrating superior accuracy and confirming the effectiveness of the proposed method.

For the conventional study based on patented technology, the accuracy of the test is not publicly available [6]. However, the proposed method addresses the limitation of not being able to detect screw threads in the second and subsequent rounds, proving its effectiveness.

The conventional study which employed machine learning did not include the mouth area in the inspection scope [4]. The proposed method, however, has been shown to inspect the mouth region with the same level of accuracy as in the conventional study.

### 4.4. Experiment 2

Building on the results of Experiment 1, an experiment was conducted to validate the feasibility of inspecting images captured on a factory production line. In this experiment, a subset of new factory images was combined with the dataset from Experiment 1, and the model was retrained. The learned model was then used to verify the inspection accuracy of the factory images.

#### 4.4.1. Production Line Demonstration of Screw Thread Image

**Figure 20:**
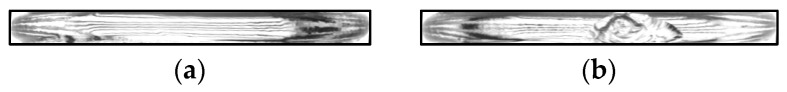
Image used for screw thread learning experiment 2 (good image (**a**), chipped image (**b**)).

The factory was able to obtain a larger number of sample bottles (300) for good products due to production, but 13 sample bottles were used for defective products, yielding 33 images. Of these 33 images, 16 were incorporated into the Experiment 1 data set for training, along with 60 good images. The trained model was then used to validate the data captured at the factory. Figure 20 provides images taken at the factory and added to the data set. Table 12 provides the details of the image data obtained at the factory prior to integration, while Table 13 presents the details of the actual data sets used.

#### 4.4.2. Experimental Results 2 (Screw Thread)

The accuracy of the model trained on the acquired screw thread images was evaluated using 1682 images for validation. Table 14 shows the confusion matrix of the detection results.

#### 4.4.3. Mouth Image Production Line Demonstration Test

**Figure 21:**
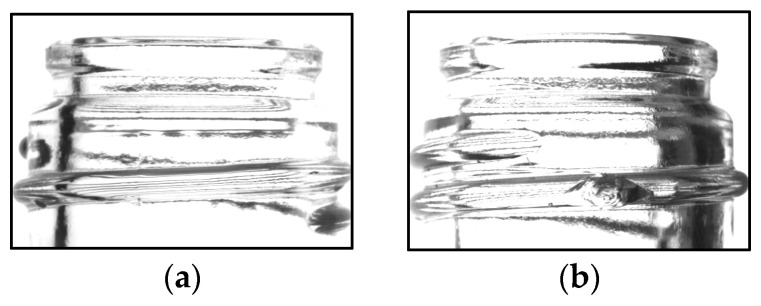
Mouth image used for experiment 2 (good image (**a**), chipped image (**b**)).

For comparison, the factory-obtained data were also integrated and used to train the model on images of the entire mouth area to assess its accuracy. Figure 21 provides images taken at the factory and added to the data set. Table 15 outlines the details of the factory-obtained image data prior to integration, while Table 16 presents the details of the actual dataset utilized.

#### 4.4.4. Experimental Results 2 (Mouth of the Bottle Image)

Accuracy was tested against a model trained on images of the entire mouth area, evaluating 1174 images for validation. The confusion matrix of detection results is shown in Table 17.

#### 4.4.5. Comparison of Experimental Results 2

Table 18 summarizes the inspection accuracy using the entire mouth and the threads only.

#### 4.4.6. Discussion of Experimental Results 2

The experimental results demonstrated that the model trained on screw thread images produced no false positives or negatives for either good or defective products, achieving a 100% accuracy rate. In contrast, the model trained on entire mouth images detected three false positives for good products, resulting in an accuracy rate of 99.7%. These findings confirm the effectiveness of the proposed method, as it improves accuracy by 0.3% compared to the full-mouth image approach. In the entire mouth image analysis, defect precision was 85.0%, the lowest recorded metric, partly due to the limited number of defective bottles tested. To improve accuracy, we plan to increase the number of test images of defective bottles in future experiments. Furthermore, the results indicate that the proposed system is suitable for detecting chipping in screw threads within a factory line.

### 4.5. About the Number of Sheets in the Data Set

In Experiment 1 of this study, 100 good bottles and 39 defective bottles were used, resulting in 640 good and 668 defective bottle images. Through data augmentation, the dataset was expanded to approximately 14,000 images for training. The inspection accuracy was high, achieving 99.7%.

In Experiment 2, an additional 319 good and 176 defective bottle images obtained from the factory were incorporated. The inspection accuracy remained at 99.7% when validated using factory-acquired images; however, the precision for defective bottles was relatively low at 85%, likely due to the limited number of defective bottle images available.

In both experiments, the training model utilizing only the screw threaded section demonstrated a significant improvement in accuracy compared to the model trained on the entire mouth, despite having a smaller training dataset. This suggests that restricting the inspection area to the threaded section can enhance inspection accuracy, even with a limited number of training images.

For future research, we plan to conduct further experiments in the factory to collect a larger dataset of bottle images and enhance inspection accuracy.

## 5. Conclusions

In this study, we proposed a method for detecting chips in the mouth of glass bottles using machine learning. In recent years, Japanese glass bottles for cosmetics have been attracting attention from the perspective of environmental friendliness due to improved manufacturing technology and the use of recycled glass, and the amount of glass bottles exported overseas has been increasing. Although glass bottles for cosmetics are required to undergo strict quality inspections from a safety perspective, the complicated shape of the glass bottle mouth makes automated inspections difficult, and visual inspections have been the norm. Manual visual inspection by workers presents challenges due to inconsistencies in judgment criteria among individuals and a decline in inspection accuracy over extended working hours. To address these issues, glass bottle inspection systems utilizing image processing and machine learning have been actively developed in recent years. Conventional image processing-based inspection methods can detect chips in glass bottles; however, they are limited to bottles without screw threads. In the glass bottles examined in this study, light from the illumination source undergoes diffuse reflection due to the presence of screw threads, leading to reduced detection accuracy. Additionally, in machine learning-based inspection methods, the evaluation is typically restricted to the body and bottom of the bottle, excluding the mouth area from analysis. To overcome these limitations, this study proposes a method that extracts screw thread regions from bottle images, develops a machine learning model, and performs defect detection based on the extracted features.

To evaluate the effectiveness of the proposed method, its accuracy was assessed by training models using images of the entire mouth and screw threads separately. The experimental results showed that the model trained on entire mouth images achieved an accuracy of 98.0%, whereas the model trained on screw thread images achieved an accuracy of 99.7%, indicating a 1.7% improvement in accuracy with the proposed method. In an experiment using factory-acquired data, the model trained on entire mouth images achieved 99.7% accuracy, while the model trained on screw thread images achieved 100%, confirming the feasibility of the proposed system for detecting chips in a factory setting. We have shown that this method can be used to detect defects in glass bottles with consistently high accuracy, regardless of human concentration.

## 6. Future Plans

In this experiment, the imaging and inspection systems operated independently because the accuracy was verified only for the images captured. In the future, we would like to integrate imaging and inspection systems, increase the number of defective samples to improve inspection accuracy, and expand the data set by generating defective bottles using GAN. In addition, we aim to handle various cases of the same type of bottles with chips on the opposite side, as well as to study the possibility of inspecting various types of glass bottles.

## Figures and Tables

**Table 1:** Accuracy of each model in fruit classification [8].

Models	Precision	Recall	F1-Score
AlexNet	0.81	0.79	0.83
VGG16	0.79	0.80	0.80
Inception V3	0.84	0.82	0.81
ResNet	0.82	0.81	0.82
MobileNetV2	0.89	0.91	0.89

**Table 2:** Finetuned hyperparameters (* epoch may vary due to early stopping).

Parameters	Value
Epochs	500 *
Optimizer	Adam
Learning rate	0.00001
Batch size	16

**Table 3:** Details on early stopping.

Parameters	Value
Monitoring metric	Validation Loss
Patience	20
Initiation epoch	100

**Table 4:** Data augmentation techniques used in the system.

Data Augmentation Mode	Changes
Random flipping	Randomly flips the image horizontally.
Random rotation	Rotates the image between −3° and 3° so that the features of the glass bottle are not lost.
Grayscale	Grayscales the image and reduces the number of features.
Filtering	Applies a bilateral filter to the image to remove noise.
Change color space	Changes the color space.
Contrast	Changes the brightness of the image.

**Table 5:** Equipment used in the experiment.

Component Parts	Specification	
Camera	Manufacturer	Baumer, Frauenfeld, Switerland
	Model number	VCXU-32M
	Resolution	2048×1536
Lens	Manufacturer	VS TECHNOLOGY, Tokyo, Japan
	Model number	VS-1218VM
Filter	Manufacturer	ZOMEI, Shenzhen, China
	Model number	ZOMEI IR850
Infrared backlight	Manufacturer	Leimac, Siga, Japan
	Model number	IFD-300/200IR-850
Light source power supply	Manufacturer	Leimac, Siga, Japan
	Model number	IWDV-300S-24

**Table 6:** Detail of image of glass bottle screw threads used in experiment 1.

	Images for Training	Images for Verification
superior article	3520	480
defective product	3608	141
total (number)	7128	621

**Table 7:** Confusion matrix of experimental results 1 (screw thread image).

	Prediction	Superior Article	Defective Product
Correct			
Superior article		480	0
Defective product		2	139

**Table 8:** Details of the image of the mouth of the glass bottle used in experiment 1.

	Images for Training	Images for Verification
superior article	7040	298
defective product	7348	244
total (number)	14,388	542

**Table 9:** Confusion matrix of experimental results 1 (mouth of the bottle image).

	Prediction	Superior Article	Defective Product
Correct			
Superior article		298	0
Defective product		11	233

**Table 10:** Evaluation of results 1 (screw threads image and mouth of the bottle image).

		Screw Thread Image	Mouth Image
Superior article	Precision	99.6%	96.4%
	Recall	100%	100%
	F1 Score	99.8%	98.2%
Defective product	Precision	100%	100%
	Recall	98.6%	95.5%
	F1 Score	99.3%	97.7%
Detection accuracy		99.7%	98.0%

**Table 11:** Comparison with previous studies (* previous research [4] does not distinguish between good and bad products in the inspection confusion matrix).

		Previous Research (Image Processing) [3,7]		Previous Research * (Machine Learning) [4]	Proposed Method	Entire Mouth Image
Superior article	Precision	98.0%	98.0%	98.8%	99.6%	96.4%
	Recall	98.0%	100%	99.3%	100%	100%
	F1 Score	98.0%	99.0%	99.0%	99.8%	98.2%
Defective product	Precision	98.0%	100%	-	100%	100%
	Recall	98.0%	98.0%	-	98.6%	95.5%
	F1 Score	98.0%	99.0%	-	99.3%	97.7%
Detection accuracy		98.0%	99.0%	98.1	99.7%	98.0%

**Table 12:** Detail of image of glass bottle screw threads used in experiment 2 (images taken at the factory).

	Images for Learning (Factory Shooting)	Images for Verification
superior article	319	1665
defective product	176	17
total (number)	495	1682

**Table 13:** Detail of image of glass bottle screw threads used in experiment 2 (dataset from Experiment 1 combined with images taken at the factory).

	Images for Learning
superior article	3839
defective product	3784
total (number)	7623

**Table 14:** Confusion matrix of experimental results (screw thread image).

	Prediction	Superior Article	Defective Product
Correct			
Superior article		1665	0
Defective product		0	17

**Table 15:** Details of the image of the mouth of the glass bottle used in experiment 2 (images taken at the factory).

	Images for Learning (Factory Shooting)	Images for Verification
superior article	660	1157
defective product	176	17
total (number)	836	1174

**Table 16:** Details of the image of the mouth of the glass bottle used in experiment 2 (dataset from Experiment 1 combined with images taken at the factory).

	Images for Learning
superior article	7700
defective product	7524
total (number)	15,224

**Table 17:** Confusion matrix of experimental results (mouth of the bottle image).

	Prediction	Superior Article	Defective Product
Correct			
Superior article		1154	3
Defective product		0	17

**Table 18:** Evaluation of results 2 (screw threads image and mouth of the bottle image).

		Screw Thread Image	Mouth Image
Superior article	Precision	100%	100%
	Recall	100%	99.7%
	F1 Score	100%	99.8%
Defective product	Precision	100%	85.0%
	Recall	100%	100%
	F1 Score	100%	91.9%
Detection accuracy		100%	99.7%

## Data Availability

The raw data supporting the conclusions of this article will be made available by the authors on request.
